# Agriculturally Sourced Multidrug-Resistant *Escherichia coli* for Use as Control Strains

**DOI:** 10.3390/pathogens14050417

**Published:** 2025-04-25

**Authors:** James E. Wells, Lisa M. Durso, Abasiofiok M. Ibekwe, Jonathan G. Frye, Manan Sharma, Clinton F. Williams, Md Shamimuzzaman

**Affiliations:** 1 USDA Agricultural Research Service (ARS), U.S. Meat Animal Research Center, Meat Safety and Quality, Clay Center, NE 68933, USA; 2 USDA Agricultural Research Service (ARS), Agroecoystem Management Research, Lincoln, NE 68583, USA; lisa.durso@usda.gov (L.M.D.); md.shamimuzzaman@usda.gov (M.S.); 3 USDA Agricultural Research Service (ARS), Agricultural Water Efficiency and Salinity Research Unit, Riverside, CA 92507, USA; mark.ibekwe@usda.gov; 4 USDA Agricultural Research Service (ARS), U.S. National Poultry Research Center, Poultry Microbiological Safety and Processing Research Unit, Athens, GA 30605, USA; jonathan.frye@usda.gov; 5 Environmental Microbial and Food Safety Laboratory, USDA Agricultural Research Service (ARS), Beltsville, MD 20705, USA; manan.sharma@usda.gov; 6 USDA ARS U.S. Arid Land Agricultural Research Center, Water Management and Conservation Research Unit, Maricopa, AZ 85377, USA; clinton.williams@usda.gov

**Keywords:** antibiotic resistance, *Escherichia coli*, agriculture, extended-spectrum β-lactamase (ESBL), CTX-M, tetracycline, *tet*(A), antimicrobial resistance, control strains, surveillance

## Abstract

Bacteriological control strains with known characteristics ensure consistency and reproducibility of assay performance across different laboratories and are an important cornerstone of quality control in the microbiology laboratory. Ideally, control strains should be representative of the assay target and be widely available from reputable sources. However, for work involving antibiotic resistance most controls come from human and veterinary clinical sources and are not optimized for work in agriculturally impacted environments or not widely available. The objective of this work was to identify and make widely available two *E. coli* isolates sourced from agricultural production settings that could be used as external controls supporting method development, research and environmental monitoring for extended spectrum β-lactamase producing (ESBL) and tetracycline resistant *Escherichia coli*. Previously collected *E. coli* suspects were screened based on antimicrobial susceptibility testing data, then confirmed as *E. coli* and characterized both phenotypically and genotypically. The positive control strain, ARS-C301 was ESBL positive and contained the CTX-M-55 and *tet*(A) genes, and the negative control strain, ARS-C101 was negative for both targets. Here we introduce two agriculturally sourced, fully characterized, and genetically sequenced control strains for use as laboratory controls in research involving extended-spectrum β-lactamase producing (ESBL) and tetracycline-resistant *Escherichia coli* isolated from the environment, available via publicly accessible culture collections, and commercially as a quantitative pellet.

## 1. Introduction

Antibiotic resistant bacterial infections continue to threaten human and animal health and critical knowledge gaps remain regarding the role of the environment as a reservoir and transport vehicle for antibiotic resistant bacteria, and the potential risks to human, animal, and ecosystem health associated with agricultural and environmental antibiotic resistance [1,2]. Soil is a natural source of antibiotic resistant bacteria [3,4,5,6] and evolutionary studies verify that antibiotic resistance in clinical pathogens arose from bacteria living in soil [7,8]. Antibiotic resistance is ubiquitously present in soils, waterways, and air, across geographies and biomes [8]. Notably, land application of manures and biosolids, fecal contributions from wildlife, and wastewater from hospitals, agriculture, and manufacturing add to the community of antibiotic resistant bacteria in the environment [2,9,10].

The use of *E. coli* as an indicator organism of fecal contamination serves as the foundation for both national and international water quality monitoring and regulatory actions [11]. *Escherichia coli* is a common fecal indicator bacteria associated with the gastrointestinal tract (GIT) of humans and animals. The *E. coli* life cycle involves both residence in the GIT and cycling through the environment external to the gut [12], although there is evidence that some *E. coli* can become naturalized in the soil [13,14]. It is the association of *E. coli* with fecal material, and their ease of culture in the laboratory, that has propelled them to prominence as an indicator organism for environmental quality monitoring. As interest in environmental antibiotic resistance grows, researchers are starting to expand the role of *E. coli* as a fecal indicator organism, to include *E. coli* as an indicator organism for monitoring antibiotic resistance [15,16]. Additionally, global antibiotic resistance control efforts seek to “anchor” monitoring efforts with existing indicator organisms such as *E. coli* [17], due in large part to the existing framework of *E. coli* used for water quality assessments and to “link” environmental antibiotic resistance to clinically relevant human health outcomes. Thus, *E. coli* is increasingly being used as an indicator for environmental monitoring of antibiotic resistance [18,19],

One desirable condition for control strains is that they represent the target of an assay. However, there is marked niche and genetic variation across *E. coli*. While most *E. coli* are harmless commensal organisms, a subset of *E. coli* are important zoonotic pathogens. Human and non-human associated *E. coli* isolates each have different phylogenies, and potentially different virulence profiles [20,21]. Virulent *E. coli* predominantly fall into groups B2 and D while commensal (non-pathogenic) *E. coli* are generally classified in groups A and B1 [22]. Even within pathogenic serotypes such as *E. coli* O157:H7, there is a phylogenetic dichotomy between human and non-human isolates, suggesting biological differences in the ability to be transmitted to and/or infect people [23,24,25].

Recent global antibiotic resistance work highlights the need for standardized methods and quality control measures [26], however the majority of antibiotic resistant isolates available for use as control strains for methods development, research, and quality control come from hospital and human public-health laboratories, such as the CDC Isolate bank https://www.cdc.gov/drugresistance/resistance-bank/index.html (accessed on 15 August 2023). In the spirit of the One Health approach to addressing antibiotic resistance and, in the context of quantitative methodology and risk assessment, there are important differences between antibiotic resistance in the clinic, and antibiotic resistance in agricultural ecosystems [2,27]. In the clinic there is a direct, evidence-based connection between the isolate, virulence, and antibiotic resistance. Conversely, most soil-, water-, and airborne bacteria are non-pathogenic. Those that are potential pathogens need to contact and infect a host (person or animal), cause illness, and be non-responsive to the treatment drug before being confirmed as having both the genotypic and phenotypic equivalent of the clinical isolates [28,29]. Environmental bacteria have, however, been shown to be the source of clinically important genes encoding resistance to β-lactams, aminoglycosides, amphenicols, sulfonamides, tetracyclines, and other antimicrobials [7,30]; and there is a growing awareness that sustainable progress in measuring antibiotic resistance in the clinic is linked to measuring and mitigating it in the natural and agricultural environment [1,8].

However, there is an inherent variability when working with living organisms that requires a unique approach to methodological and quality control. Bacteriological control strains serve to provide rigor to experimental results by providing information that helps to ensure that data are accurate. Bacterial control strains are needed to ensure consistency and reproducibility for a wide range of analytical procedures and processes tailored to agricultural and environmental settings [31]. In addition to the widely used standard control strains required as part of clinical antibiotic resistance susceptibility testing, control strains are also essential when working to detect, quantify, and isolate bacteria in water, soil, and air. They can ensure that microbial enumeration is accurate and that results are consistent across locations, providing trusted information for policy makers and producers to make data-driven decisions [32]. Criteria for selecting control strains include having a well-documented origin, thorough phenotypic and genotypic typing, deposition and long-term storage in accredited biobank, ensuring strains are readily available, and minimizing propagation passages [33]. Given these requirements and in support of One Health efforts, there is a need for broadly accessible non-clinical, non-human associated *E. coli* controls strains to monitor environmental assay and performance quality, separate from the controls used for the interpretation of bacterial susceptibility in clinical laboratories. The goals of the current work were (1) to identify agriculturally sourced positive and negative *E. coli* strains with target antibiotic resistance phenotypes for use as quality controls in research and method development, and (2) to make the isolates publicly available to the research community.

## 2. Materials and Methods

### 2.1. Initial Isolate Screening

A set of agriculturally associated *E. coli* isolates were screened for their utility as extended spectrum β-lactamase producing (ESBL)/cefotaxime resistant/tetracycline resistant control strains, in support of One Health laboratory methods. ESBL resistance was chosen as a target to align with U.S. [34] and global health priorities [29], including the World Health Organization (WHO) Tricycle surveillance protocol [17], as well the new National Antibiotic Resistance Monitoring System (NARMS) environmental surveillance effort [19]. Tetracycline resistant *E. coli* were chosen as a second target because the tetracyclines are the most widely used antibiotic in food animal production both globally and in the U.S.; and tetracycline is the most commonly studied resistance type in environmental and agricultural samples [35,36].

Enteric isolates were previously collected from cattle using antibiotic-enriched CHROMAgar *E. coli* (DRG International, Mountainside, NJ, USA) media with and without 2 mg/L of cefotaxime, 4 mg/L trimethoprim and 76 mg/L sulfamethoxazole, or 32 mg/L tetracycline [37]. As described in Long et al. [37] antibiotic sensitivity testing was performed using the Sensititre broth microdilution CMV4AGNF test plates (Thermo Scientific, Waltham, MA, USA) and the Sensititre ARIS HiQ System (Thermo Scientific) for 14 antibiotics from 13 drug classes (Table 1), and the NARMS CDC breakpoints (amoxicillin–clavulanic acid (≥32 μg/mL), ampicillin (≥32 μg/mL), azithromycin (≥32 μg/mL), cefoxitin (≥32 μg/mL), ceftriaxone (≥4 μg/mL), chloramphenicol (≥32 μg/mL), ciprofloxacin (≥1 μg/mL), gentamicin (≥16 μg/mL), nalidixic acid (≥32 μg/mL), streptomycin (≥32 μg/mL), sulfisoxazole (≥512 μg/mL), tetracycline (≥16 μg/mL), and trimethoprim–sulfamethoxazole (≥4 μg/mL) [37].

### 2.2. Isolate Selection and Phenotypic Confirmation

The Long et al. [37] *E. coli* Antimicrobial Susceptibility testing (AST) data was screened manually to identify isolates that displayed susceptibility or resistance to second and third generation cephalosporins and/or tetracycline. A subset was chosen for confirmatory testing and revived from freezer stocks. *E. coli* was confirmed using a standard biochemicals, differential media, *uidA* PCR and 16s rDNA sequencing, then screened for ESBL production. Isolates were sequenced prior to deposition in accessible culture collection.

More specifically, isolates were confirmed as *E. coli* using the indole test in tryptone broth (US Biological, Salem, MA, USA) with Kovac’s reagent (Hardy Diagnostics, Santa Maria, CA, USA), and via morphology on the differential CHROMagar ECC media (DRG International, Springfield, NJ, USA) and Tryptone Bile X-glucuronide (TBX) agar (Fisher Scientific, Waltham, MA) with and without cefotaxime (4 µg/mL) (ctx4) (GBiosciences, St. Louis, MO, USA) and tetracycline (32 µg/mL) (tet32) (Sigma Aldrich, Burlington, MA, USA). TBX was used to align with the WHO Tricycle protocol [17]. The higher than standard tetracycline concentration was based on preliminary data showing a discrepancy in field isolations between growth on a plate containing 16 µg/mL tetracycline, and CLSI disc diffusion results (unpublished data).

ESBL phenotypes were assessed using both the CLSI combination disc diffusion test (CDT) [39], and the EUCAST double disc synergy test (DDST) [40]. A detailed protocol is available via Protocols IO [41] Both tests were conducted using Mueller Hinton (MH) broth (HiMedia, Kennett Square, PA, USA) and agar (BD, Franklin Lakes, NJ, USA). Overnight cultures were spun down and resuspended in 100 µL of phosphate buffered solution (PBS) (Fisher, Waltham, MA, USA). The suspension was adjusted to a 0.5 McFarland standard, and evaluated using a spectrophotometer with a reading between 0.08–0.13 at 600 nM. The CDT used cefotaxime 30 µg (CTX), cefotaxime/Clavulanic Acid 30/10 µg (CTX/CA), ceftazidime 30 µg (CAZ), and ceftazidime/Clavulanic Acid 30/10 µg (CAZ/CA) discs (BD, Franklin Lakes, NJ, USA). Following inoculation of the plate, discs were placed roughly in quadrants, approximately 22 mm apart. Plates were incubated for 18 ± 2 h at 35 °C ± 2 °C. Calipers, measuring in mm, were used to measure the zone of inhibition (ZOI) around each of the antibiotic discs. CDT results were considered ESBL positive if there was ≥5 mm size difference in the zone of inhibition between the single drug and the drug plus clavulanic acid (CA). The DDST discs consisted of cefotaxime 30 µg, amoxicillin/Clavulanic Acid 30 µg (AMC) and ceftazidime 30 µg. On the DDST plate the discs were arranged linearly with the AMC disc in the middle, then incubated and measured as above. DDT results were considered positive if the ZOI was larger on the AMC side for both CTX and CAZ halos, with a bowtie or barbell shape indicating a negative test.

Finally, confirmed isolates were evaluated for phenotype stability using the patch or replica-plate method [42] on Tryptic soy Agar (TSA) (BD Difco, Franklin Lakes, NJ, USA) with and without ctx4 and tet32. A sterile inoculating needle was used to dip into an isolated colony and then dab the needle onto the media types, using a different needle between media types and antibiotics [42]. The plates were incubated overnight at 37 °C and scored visually for presence/absence of growth. This process was completed for 20 passages.

### 2.3. Genotypic Confirmation

Control strain isolates were subjected to confirmatory PCR using the *E. coli uidA* gene [43]. Briefly, DNA was extracted using the boil method. Reactions consisted of 12.5 µL RedTaq ReadyMix (Sigma, St. Louis, MO, USA), 7.3 µL HyClone PCR Water (Sigma, St. Louis, MO, USA), 0.1 µL (100 µM) forward primer *uidA* F241 (5′-CAGTCTGGATCGCGAAAACTG-3′), 0.1 µL (100 µM) reverse primer *uidA* R383 (5′-ACCAGACGTTGCCCACATAATT-3′) (IDT, Coralville, IA, USA), and 1 µL DNA template. Thermocycling conditions consisted of 1 cycle at 95 °C for 1 min; 40 cycles at 94 °C for 10 s, 63 °C for 40 s, 72 °C for 30 s; and 1 cycle at 72 °C for 5 min. Amplicons were electroporated on a 1% agarose gel and imaged using a Biorad Gel Doc XR+ (BioRad, Hercules, CA, USA) [44]. Phylotyping was performed as described previously [45], using the Doumith et al. [46] modification of the Clermont method [47], and the Escobar-Paramo phylotyping scheme [48]. Isolate DNA was also shipped to Biolog-MIDI Lab (Newark, DE, USA) for confirmatory 16S sequencing.

### 2.4. Whole Genome Sequencing

Isolates were grown overnight in tryptic soy broth (TSB) (Difco, Franklin Lakes, NJ, USA), pelleted and washed twice with phosphate buffered saline, then suspended in DNA Shield (Zymo, Irving, CA, USA) and shipped overnight to Plasmidsaurus (Eugene, OR, USA) for whole genome sequencing using the Nanopore platform. A long-read sequencing library was constructed using Nanopore v14 library prep chemistry (Oxford Nanopore Technologies, Oxford, UK) and run on an Oxford Nanopore R10.4.1 flow cell. The output was assembled using Flye [49] v2.9.1, polished using Nanopore Medaka v1.8.0, and annotated using Bakta v1.6.1 [50]. The quality of genome assemblies was assessed using CheckM (v1.2.2) with default parameters [51]. It uses a set of reference gene profiles to assess the completeness and contamination. This displays the contigs which the specific CheckM reference genes were found. The reference genes are almost always chromosomal, so a contig plot mostly shows the chromosomal references genes. Mash (v2.3) [52] was utilized to find the identity between assembled contigs against RefSeq database of bacterial genomes and plasmids. Genome wide antibiotic resistance genes (ARGs) were identified using the Comprehensive Antibiotic Resistance Database (CARD) Resistance Gene Identifier (RGI 6.0.3) [53] with default settings and maps were created using Proksee [54].

## 3. Results

### 3.1. Screening and Phenotypic Characterization

Of the 1133 *E. coli* suspects collected with AST data available [37], 192 were scored as resistant to cefoxitin, ceftriaxone, or tetracycline; 44 were scored as susceptible to all three drugs and 17 were scored as susceptible to all 14 drugs tested. A subset of 29 isolates with either dual cefoxitn/tetracycline resistant phenotype or pan-susceptible phenotype were chosen for further characterization—an initial set of eight, and a subsequent set of 21. Of these 29 isolates, five were negative for the indole reaction and were excluded from further consideration. The top candidates were evaluated for growth on TSA and TBX with and without cefotaxime and tetracycline, as described below. All 17 isolates gave expected results that aligned with the Long et al. [37] Sensititre AST data. Two of the isolates were ESBL positive by the CDT disc diffusion assay.

**Figure 1:**
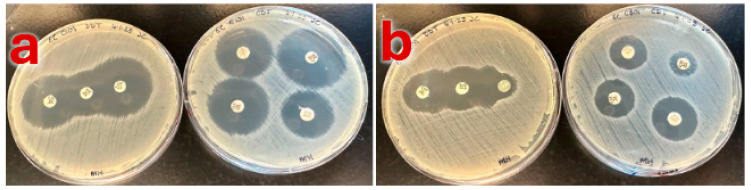
(**a**) ARS-C101: Double Disc Synergy Test (Left) and Combination Disc Diffusion Test (Right). (**b**) ARS-C301: Double Disc Synergy Test (Left) and Combination Disc Diffusion Test (Right).

Based on the initial screening, isolate ARS-C301 was tentatively selected as the positive control, and ARS-C101 was tentatively selected as the negative control (Table 2). Both were collected in 2021 [37] from cattle fecal samples in Texas. ARS-C301 was originally isolated from a microbiological plate with added Trimethoprim–sulfamethoxazole, and there was no selective antibiotic used on the plate from which ARS-C101 was isolated [37]. MIC values for two control strains are listed in Table 1. Both isolates were indole positive, grew typical blue-green colonies on TBX agar, and blue colonies on CHROMagar ECC. ESBL results are provided in Table 3 and Table 4, and Figure 1. ARS-C301 was positive and ARS-C101 negative for ESBL production by both the CLSI and EUCAST methods. Of note, the U.S. based CLSI only recommends the CDT, not the DDST.

### 3.2. Genotypic Characterization

Both ARS-C301 and ARS-C101 were genotypically confirmed as *E. coli E. coli* via 16S sequencing of the V1 and V2 regions (Biolog-MIDI Lab, Newark, DE, USA). The ARS-C301 sequence aligned with an Irish soil *E. coli* partial sequence (GenBank: GQ273520.1), and the ARS-C101 sequence most closely matched a clinical *E. coli* O136:H- partial sequence from Japan (GenBank: AB604195.1). Both control strains were phylotyped by PCR [45,46,47], and classified as belonging to the B1 group, being positive for *uidA*, gadA, and TSPE4.C2, and negative for *chuA* and *yjaA*.

### 3.3. Assembly and Annotation of Two Agriculturally Sourced E. coli Genomes

**Figure 2:**
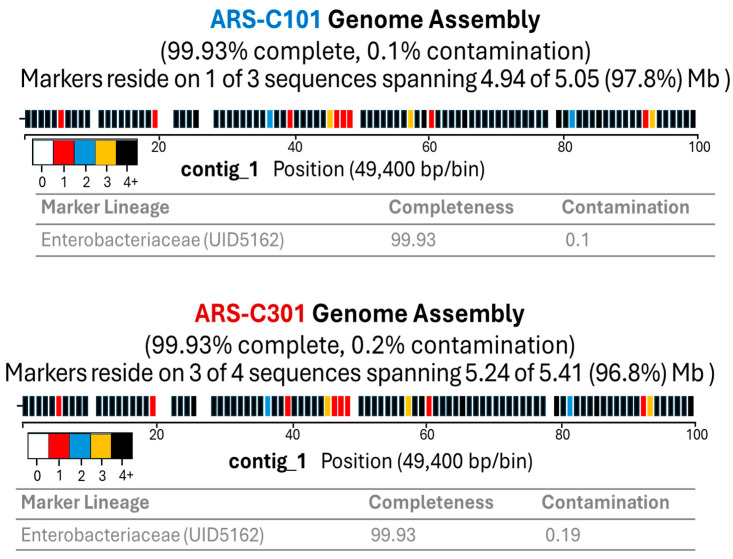
Genome assembly completeness.

The nanopore raw reads, 108,488 (ARS-C101) and 130,182 (ARS-C301), were used to assemble genomic contigs using Flye [49] v2.9.1. The resulting assembly provides genome coverage of 98× and 108× for ARS-C101 and ARS-C301 respectively (Table 5). We assembled ARS-C101 genomes into 3 contigs and the longest contig is 4,937,799 bp long whereas ARS-C301 genome was assembled into 4 contigs with 4,894,443 bp longest contig. The genome annotation using Bakta v1.6.1 generated 5041 and 5432 genes for ARS-C101 and ARS-C301 genome respectively (Table 5). The genome completeness and contamination were assessed using CheckM tools. It shows the genomic contig bins with the presence and absence of reference marker genes using small colored rectangle having different frequencies (Figure 2). Our CheckM results showed 99.93% completed genomes with 0.1% and 0.19% contamination for ARS-C101 and ARS-C301 genome respectively (Figure 2).

### 3.4. Finding Best Hits for Assembled Genomes Using Mash

A rapid genomic similarity finding tool named Mash was utilized to get best hits for assembled contigs against the RefSeq database of bacterial genomes and plasmids. For ARS-C101 genome, contig1 was matched to Agriculturally sourced *E. coli* RefSeq accession NZ_JXVO01000001.1 (Table 6). Other two contigs for ARS-C101 genome matched to *E. coli* plasmids p1303_5 (accession NZ_CP009169.1) and pECC-1470_100 (accession NZ_CP010345.1). Four contigs of ARS-C301 genome was used to find the best RefSeq hits. Contig1 was matched to Agriculturally sourced *E. coli* RefSeq accession NZ_MPUD01000003.1 (Table 7). Three other contigs matched to *E. coli* plasmids p15.01CC_2 (accession AP027468.1), pVPS18EC0467-2 (accession CP063740.1) and pGSH8M-2-1 (accession AP019676.1).

### 3.5. Identification of Antibiotic Resistance Genes Using CARD-RGI

**Figure 3:**
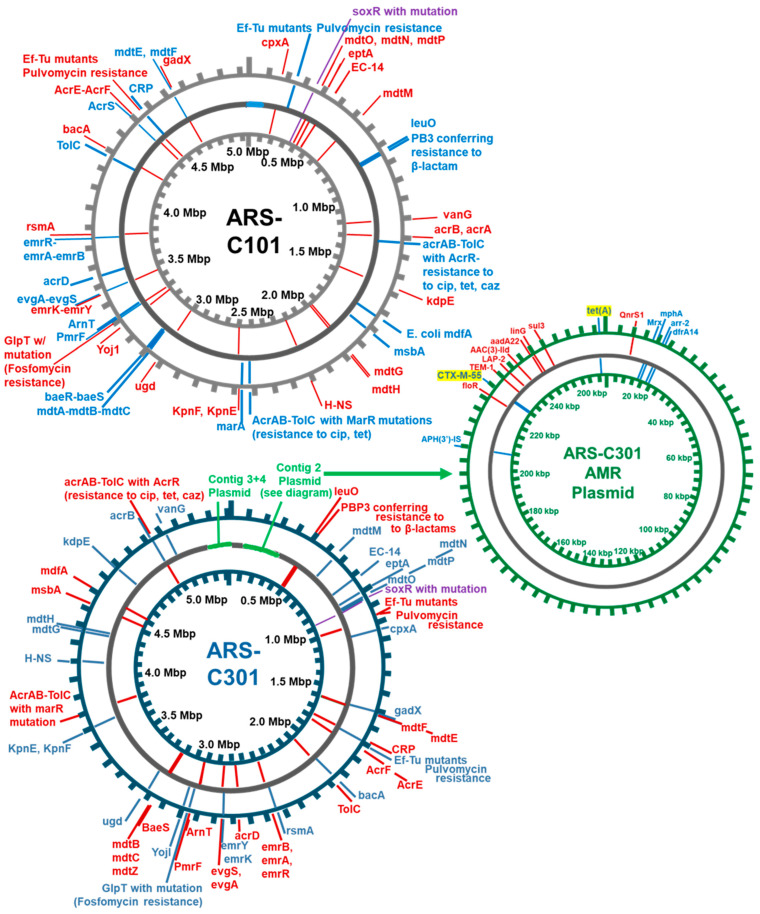
Environmental Control Strain Genome Maps Showing ARGs. ARS-C101 is the negative control strain and has an ESBL negative and tetracycline susceptible phenotype. ARS-C301 is the positive control strain and has an ESBL positive and tetracycline resistant phenotype. The inner- and outer-most circles are for orientation and to indicate gene location. The middle circle represents the genome, with grey indicating regions with full coverage. Genes located on the positive strand are marked in blue, those on the negative strand are marked in red. See also Supplementary File S1 for larger images of the maps, and Supplementary File S2 for data used to compose the figure.

ARS-C101 and ARS-C301 had 98× and 106× assembled coverage, respectively from 108,488 (ARS-C101) and 130,182 (ARS-C301) reads. Genomes were screened for antibiotic resistance genes using the Comprehensive Antibiotic Resistance Database RGI-Identifier tool [53] (Figure 3). The isolates shared 55 resistance determinants, and ARS-C301 carried an additional 15, including CTX-M-55, LAP-2, and TEM-1 supporting the ESBL phenotype, and *tet*(A) efflux pump supporting the tetracycline resistance phenotype. Additionally, resistance determinants for macrolide, lincosamide, aminoglycoside, fluroquinolone, sulfonamide, rifamycin diaminopyrimidine, and phenicol resistance were also found in the ARS-C301 genome (Supplementary Files S1 and S2). Both strains are publicly available, and the ARS-C301 strain is available as a pre-quantified pellet through Microbiologics (Saint Cloud, MN, USA) (see data availability statement below).

## 4. Discussion

The objective of this work was to identify and make widely available two *E. coli* isolates sourced from agricultural production settings that could be used in the laboratory as external controls supporting method development, research, and environmental monitoring: (1) an isolate that displayed phenotypic resistance specifically to tetracycline and cefotaxime, and was positive for ESBL production when evaluated using the Clinical Laboratory Standards Institute combination disk diffusion test [39]; (2) a negative control that was sensitive to both target antibiotics.

Since the focus of the effort was on agricultural and environmental settings and their relevance to human health outcomes, the final target selection was a compromise between clinical importance, and functionality in the context of large-scale agroecosystem and environmental monitoring efforts. Considerations included potential use of the strains for methods development and validation, as well as for research and surveillance. Given the labor- and cost-intensive nature of culture-based methods the decision was made to focus on only two targets. Although a number of clinically relevant targets were considered, the final decision to choose cefotaxime resistance was influenced primarily by the desire to align with and support the foundational work that had already been done to develop the Tricycle protocol [17].

The second target, tetracycline, is not generally considered a priority for human or veterinary antimicrobial resistance monitoring efforts. It is classified as an “important antimicrobial” by a joint commission of the Food and Agriculture Organization (FAO), the United Nations Environmental Programme (UNEP), and the World Organization for Animal Health (WOAH) [55], but the “important” classification is the lowest ranking, and tetracycline is not included in the veterinary-specific list of (clinically) important antimicrobials. However, tetracyclines are still broadly used in human and veterinary medicine to treat a variety of infections, such as gonorrhea, in humans [34]. Tetracyclines are also widely used in food animal production and veterinary care of pets, making them a relevant target given the project goals. Tetracyclines account for 37% of all veterinary antibiotics sold in 2023 and represent the largest amount (66%) of any veterinary drug also used in human medicine [56]. Tetracycline resistance has also been a keystone target for surveillance of antibiotic resistance in agricultural and environmental systems, and there is a large body of research that supports the utility of tetracycline gene targets for understanding the fate and transport of antibiotic resistance in these settings [26].

There are a number of fundamental differences between antibiotic resistance monitoring efforts in human and veterinary clinical settings compared to the environment [27] that additionally influenced the decision to choose tetracycline resistance as the second target for the control strains. First, the laboratory infrastructure is different in clinical compared to environmental antimicrobial resistance monitoring. There is a well established network of clinical diagnostic laboratories associated with and supported by the health care system, supplemented and coordinated by government funded public health laboratories. In contrast environmental antimicrobial resistance monitoring is generally performed ad hoc by independent academic and research laboratories. On top of the differences in infrastructure and scale, clinical monitoring starts with a patient who is already sick enough to seek healthcare, which serves as a concentration mechanism to increase the likelihood of finding an antibiotic resistant organism [1,27]. In natural and agricultural settings such as soil and water, the majority of the bacteria are not pathogens, and the likelihood of finding an organism carrying a medically important type of antibiotic resistance is extremely low. While theoretically having a large scale, long-term monitoring effort that results in all or most samples being negative can be useful and interesting information, to be useful, environmental monitoring of antibiotic resistance needs to be focused [2], and a minimum number of positives are required for the data to be informative from the perspective of ongoing risk assessment, or for understanding the ecology, fate, and transport of antibiotic resistance in agriculturally impacted and natural environments. This consideration made higher priority human and veterinary health targets less desirable for the current control strain project. Practically, it was difficult to imagine financially sustaining an expensive effort that consistently returned predominantly negative results. Thus, the proven utility of tetracycline monitoring in natural environments and its relevance to animal agriculture were the justifications for choosing tetracycline as the second target.

One intended use of these control strains is as external methodological controls in support of local, national, and global environmental antibiotic resistance surveillance efforts. In 2021 WHO released the Tricycle protocol, which includes an environmental surveillance component as part of a coordinated experimental design that also includes linked human and animal sample collection and analysis [17]. In that instance, the control strain is used both quantitatively and for quality control during membrane filtration. The Tricycle control strain used for the environmental component [57] is not currently available through ATCC or other public culture collections, limiting accessibility. While the isolates described here do not replace those currently in use for larger efforts, they do provide an easily accessible functionally similar alternative for new efforts, such as the national surface water monitoring program being undertaken by the NARMS environmental working group [19]. In addition to large-scale projects, standardized control strains such as those described here could also be useful in coordinating individual efforts across locations and sample matrices, to support the creation of data pools useful for modeling and risk assessment.

Another intended use of the isolates described here is for method development and validation efforts, providing an easily accessible, well-characterized, and agriculturally sourced alternative to clinical isolates. An important criterion for control strains is that they should be biologically representative of the assay target [33]. Since a major focus of environmental antibiotic resistance monitoring efforts is agriculture and food animal production, having agriculturally-sourced control strains provides that representation. The ARS-C301 and ARS-C101 isolates belong to phylogroup B1, which was the most abundant group among agricultural isolates in a previous study [45].

Across the One Health spectrum, there is an urgent need for improved diagnostics and methodology to provide faster, more accurate, and more accessible results to support antimicrobial resistance decision making and control efforts. This is especially true for the environmental component of One Health, which despite recent advances still lags behind its human and animal health counterparts [58]. One key feature of the control strains introduced here is that they are publicly available to researchers through multiple sources. Having the same control strain widely and easily available to all professionals eliminates one major hurdle to assay validation, supports efforts to standardize methods, and enhances the impact of individual efforts by making cross-study and cross-location comparisons more useful. Both strains are deposited with ATCC (formerly the American Type Culture Collection), and they are also available with minimal cost to researchers in the U.S. through the USDA-ARS Northern Regional Research Laboratory (NRRL) Culture Collection in Peoria, IL. Additionally, pre-quantified pellets of ARS-C301 (the positive control strain) are available for purchase through a private company (see data and strain availability section below).

## 5. Conclusions

There is a need for broadly accessible non-clinical, non-human associated *E. coli* to serve as controls strains in studies focusing on antibiotic resistance in agricultural and environmental surveillance of antibiotic resistant bacteria and antibiotic resistance method development. It is hoped that as new One Health focused efforts are undertaken, the ARS-C301 and ARS-C101 control strains will be useful to the scientific and public health community.

## Figures and Tables

**Table 1:** List of antibiotics used in antimicrobial susceptibility testing, and strain MIC values from Long et al.

		ARS-C101 (Neg)		ARS-C301 (Pos)	
	Drug	MIC	Interpretation	MIC	Interpretation
1	Amoxicillin/Clavulanic Acid	4	S	8	S
2	Ampicillin	2	S	32	R
3	Azithromycin	8	S	32	R
4	Cefoxitin	4	S	8	S
5	Ceftriaxone	0.25	S	64	R
6	Chloramphenicol	8	S	32	R
7	Ciprofloxacin	0.015	S	0.25	S
8	Gentamicin	0.5	S	16	R
9	Meropenem	0.06	S	0.06	S
10	Nalidixic Acid	4	S	4	S
11	Streptomycin	8	S	64	R
12	Sulfisoxazole	16	S	512	R
13	Tetracycline	4	S	32	R
14	Trimethoprim/Sulfamethoxazole	0.12	S	4	R

S = susceptible as scored by Sensitre, R = resistant as scored by Sensititre. Results collected and reported by Long et al. [37], using methods from Agga et al. [38].

**Table 2:** Control Strain Results and Metadata.

Isolate ID	ARS-C101	ARS-C301
Description	*E. coli*, NEGATIVE CONTROL ESBL−, CTX^S^, TET^S^	*E. coli*, POSITIVE CONTROL ESBL+, CTX^R^, TET^R^
Date Collected	7 February 2021	28 March 2021
Source	Cattle Fecal Grab	Cattle Fecal Grab
Location	Texas	Texas
*E. coli* phenotypic confirmation	Blue-green colonies on TBX agar, blue colonies on CHROMagar ECC, pink colonies on CHROMagar Orientation. Indole positive.	Blue-green colonies on TBX agar, blue colonies on CHROMagar ECC, pink colonies on CHROMagar Orientation. Indole positive.
CDT Zone Sizes	(CTX) 31.2 mm, (CTX/CA) 24.7 mm (CAZ) 27.1 mm, (CAZ/CA) 21.8 mm	(CTX) 6.6 mm, (CTX/CA) 16.8 mm (CAZ) 16 mm, (CAZ/CA) 21 mm
Combination Disk Diffusion Results	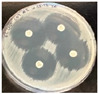	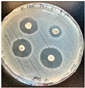
*E. coli uidA* PCR	*uidA* positive	*uidA* positive
Phylotyping via PCR	B1	B1
Target Gene Genotype	Not applicable	CTX-M-55, *tet*(A)
16S GenBank assignment (V1–V2) *	*E. coli* O136: H-(GenBank: AB604195), Japan. 528 bp	*E. coli* JCLys7 (GenBank GQ273520.1), from temperate gley soil, Ireland. 528 bp
16S sequence Accession #	OR269615	OR269616
NCBI WGS Accession #	Accession number: PRJNA1003888 BioSample: SAMN36909132	Accession number: PRJNA1003888 BioSample: SAMN36910816
ARS Culture Collection ID	https://nrrl.ncaur.usda.gov/ Accession number: B-65681 Accessed on 23 April 2025	https://nrrl.ncaur.usda.gov/ Accession number: B-65682 Accessed on 23 April 2025
ATTC Culture Collection ID	ATCC^®^ BAA-3340™	ATCC^®^ BAA-3341™
Microbiologics 10–100 CFU Pellets	Not available	ARS-C301

AMR = antimicrobial resistance. ESBL = Extended-spectrum β-lactamase. MDR = Multi Drug Resistant. AMX = Amoxicillin/Clavulanic Acid, AMP = ampicillin, AZM = azithromycin, FOX = cefoxitin, CRO = ceftriaxone, CHL = Chloramphenicol, CIP = ciprofloxacin, GEN = gentamicin, MEM = meropenem, NAL = nalidixic acid, STR = Streptomycin, SOX = sulfisoxazole, TET = tetracycline, SXT = Trimethoprim/sulfamethoxazole. * As reported by Biolog-Midi lab, Newark, NJ, USA. # = number.

**Table 3:** ESBL Combination Disc Test Results (mm) for ARS-C301 and ARS-C101.

Isolate	CTX	CTX/CA	Difference	CAZ	CAZ/CA	Difference	ESBL?
ARS-C301	6.6	16.8	10.2	16.0	21.0	5.0	Yes
ARS-C101	32.5	32.6	0.1	28.7	29.1	0.3	No

**Table 4:** ESBL Double Disc Test (DDT) results for ARS-C301 and ARS-C101.

Isolate	CTX	AMC	CAZ	ESBL?
ARS-C301	12.8	21.6	18.9	Yes
ARS-C101	33.5	24.8	29.8	No

**Table 5:** Summary Statistics of Sequenced *E. coli* Genomes.

	ARS-C101	ARS-C301
Total bp sequenced	703,796,709 bp	866,611,913 bp
Total number of reads	108,488 reads	130,182 reads
Longest read	100,534 bp	107,946 bp
Raw coverage	139×	160×
Assembled coverage	98×	106×
Genome size (Mb)	5.0 Mb	5.4 Mb
Number of contigs	3 contigs	4 contigs
Number of genes annotated	5041 genes	5432 genes

**Table 6:** Assembled contigs of ARS-C101 Genome with best hits against NCBI RefSeq.

Contig	Size (bp)	Identity (% Matched)	NCBI Accession	Description
Contig_1	4,937,799	99.8	NZ_JXVO01000001.1	*Escherichia coli* strain OLC-157 Cont0001
Contig_2	9686	100.0	NZ_CP009169.1	*Escherichia coli* 1303 plasmid p1303_5
Contig_3	102,406	99.2	NZ_CP010345.1	*Escherichia coli* ECC-1470 plasmid pECC-1470_100

**Table 7:** Assembled contigs of ARS-C301 Genome with best hits against NCBI RefSeq.

Contig	Size (bp)	Identity (% Matched)	NCBI Accession	Description
Contig_1	4,894,443	99.90	NZ_MPUD01000003.1	*E. coli* strain K30 IMT32646_S7_L001_R1_contig_1
Contig_2	267,517	99.92	AP027468.1	*E. coli* 15.01CC plasmid p15.01CC_DNA
Contig_3	171,722	100.00	CP063740.1	*E. coli* strain 18SC04V04-Ec plasmid pVPS18EC0467-2
Contig_4	81,040	98.54	AP019676.1	*E. coli* GSH8M-2 plasmid pGSH8M-2-1DNA

## Data Availability

ARS-C101 (negative control): WGS is available from NCBI, accession number PRJNA1003888, BioSample B-65681; Isolate is available from the ARS Culture Collection https://nrrl.ncaur.usda.gov/ (Accession number: B-65681); and from ATCC (isolate ID BAA-3340). ARS-C301 (positive control): WGS is available from NCBI, accession number PRJNA1003888, BioSample SAMN36910816; Isolate is available from the ARS Culture Collection https://nrrl.ncaur.usda.gov/ (Accession number: B-65682); from ATCC (isolate ID BAA-3341); and from Microbiologics as a quantitative pellet, via a custom order (contact industrial microbiology QC products regional sales manager by email requesting a quote for ARS-C301 https://www.microbiologics.com/contact-us). All links accessed 23 April 2025.

## Supplementary Materials

The following supporting information can be downloaded at: https://www.mdpi.com/article/10.3390/pathogens14050417/s1, Supplementary File S1: Map Images; Supplementary File S2: Supplementary Data.

## References

[1] Larsson D.J., Flach C.F. (2022). Antibiotic resistance in the environment. Nat. Rev. Microbiol..

[2] Bengtsson-Palme J., Abramova A., Berendonk T.U., Coelho L.P., Forslund S.K., Gschwind R., Heikinheimo A., Jarquín-Díaz V.H., Khan A.A., Klümper U. (2023). Towards monitoring of antimicrobial resistance in the environment: For what reasons, how to implement it, and what are the data needs?. Environ. Int..

[3] Allen H.K., Donato J., Wang H.H., Cloud-Hansen K.A., Davies J., Handelsman J. (2010). Call of the wild: Antibiotic resistance genes in natural environments. Nat. Rev. Microbiol..

[4] Durso L.M., Miller D.N., Wienhold B.J. (2012). Distribution and quantification of antibiotic resistant genes and bacteria across agricultural and non-agricultural metagenomes. PLoS ONE.

[5] Cytryn E. (2013). The soil resistome: The anthropogenic, the native, and the unknown. Soil Biol. Biochem..

[6] Wang F., Fu Y.H., Sheng H.J., Topp E., Jiang X., Zhu Y.G., Tiedje J.M. (2021). Antibiotic resistance in the soil ecosystem: A One Health perspective. Curr. Opin. Environ. Sci. Health.

[7] Forsberg K.J., Reyes A., Wang B., Selleck E.M., Sommer M.O., Dantas G. (2012). The shared antibiotic resistome of soil bacteria and human pathogens. Science.

[8] Wright G.D. (2010). Antibiotic resistance in the environment: A link to the clinic?. Curr. Opin. Microbiol..

[9] Ashbolt N.J., Amézquita A., Backhaus T., Borriello P., Brandt K.K., Collignon P., Coors A., Finley R., Gaze W.H., Heberer T. (2013). Human health risk assessment (HHRA) for environmental development and transfer of antibiotic resistance. Environ. Health Perspect..

[10] Williams-Nguyen J., Sallach J.B., Bartelt-Hunt S., Boxall A.B., Durso L.M., McLain J.E., Singer R.S., Snow D.D., Zilles J.L. (2016). Antibiotics and antibiotic resistance in agroecosystems: State of the science. J. Environ. Qual..

[11] EPA (2021). Factsheet on Water Quality Parameters, Escherichia coli. https://www.epa.gov/system/files/documents/2021-07/parameter-factsheet_e.-coli.pdf.

[12] Savageau M.A. (1983). *Escherichia coli* habitats, cell types, and molecular mechanisms of gene control. Am. Nat..

[13] Lang N.L., Smith S.R. (2007). Influence of soil type, moisture content and biosolids application on the fate of *Escherichia coli* in agricultural soil under controlled laboratory conditions. J. Appl. Microbiol..

[14] Jang J., Hur H.G., Sadowsky M.J., Byappanahalli M.N., Yan T., Ishii S. (2017). Environmental *Escherichia coli*: Ecology and public health implications—A review. J. Appl. Microbiol..

[15] Navarro-González N., Porrero M.C., Mentaberre G., Serrano E., Mateos A., Domínguez L., Lavín S. (2013). Antimicrobial resistance in indicator *Escherichia coli* isolates from free-ranging livestock and sympatric wild ungulates in a natural environment (Northeastern Spain). Appl. Environ. Microbiol..

[16] Agunos A., Gow S.P., Léger D.F., Carson C.A., Deckert A.E., Bosman A.L., Loest D., Irwin R.J., Reid-Smith R.J. (2019). Antimicrobial use and antimicrobial resistance Indicators—Integration of Farm-Level surveillance data from broiler chickens and turkeys in British Columbia, Canada. Front. Vet. Sci..

[17] World Health Organization (2021). WHO Integrated Global Surveillance on ESBL-Producing E. coli Using a “One Health” Approach: Implementation and Opportunities.

[18] Anjum M.F., Schmitt H., Börjesson S., Berendonk T.U., Donner E., Stehling E.G., Boerlin P., Topp E., Jardine C., Li X. (2021). The potential of using *E. coli* as an indicator for the surveillance of antimicrobial resistance (AMR) in the environment. Curr. Opin. Microbiol..

[19] Franklin A.M., Weller D.L., Durso L.M., Bagley M., Davis B.C., Frye J.G., Grim C.J., Ibekwe A.M., Jahne M.A., Keely S.P. (2024). A one health approach for monitoring antimicrobial resistance: Developing a national freshwater pilot effort. Front. Water.

[20] Ochman H., Selander R.K. (1984). Standard reference strains of *Escherichia coli* from natural populations. J. Bacteriol..

[21] Denamur E., Clermont O., Bonacorsi S., Gordon D. (2021). The population genetics of pathogenic *Escherichia coli*. Nat. Rev. Microbiol..

[22] Picard B., Garcia J.S., Gouriou S., Duriez P., Brahimi N., Bingen E., Elion J., Denamur E. (1999). The link between phylogeny and virulence in *Escherichia coli* extraintestinal infection. Infect. Immun..

[23] Kim J., Nietfeldt J., Benson A.K. (1999). Octamer-based genome scanning distinguishes a unique subpopulation of *Escherichia coli* O157: H7 strains in cattle. Proc. Natl. Acad. Sci. USA.

[24] Franz E., Rotariu O., Lopes B.S., MacRae M., Bono J.L., Laing C., Gannon V., Söderlund R., Van Hoek A.H., Friesema I. (2019). Phylogeographic analysis reveals multiple international transmission events have driven the global emergence of *Escherichia coli* O157: H7. Clin. Infect. Dis..

[25] Ketkhao P., Utrarachkij F., Parikumsil N., Poonchareon K., Kerdsin A., Ekchariyawat P., Narongpun P., Nakajima C., Suzuki Y., Suthienkul O. (2024). Phylogenetic diversity and virulence gene characteristics of *Escherichia coli* from pork and patients with urinary tract infections in Thailand. PLoS ONE.

[26] Liguori K., Keenum I., Davis B.C., Calarco J., Milligan E., Harwood V.J., Pruden A. (2022). Antimicrobial resistance monitoring of water environments: A framework for standardized methods and quality control. Environ. Sci. Technol..

[27] Durso L.M., Cook K.L. (2014). Impacts of antibiotic use in agriculture: What are the benefits and risks?. Curr. Opin. Microbiol..

[28] Stanton I.C., Bethel A., Leonard A.F.C., Gaze W.H., Garside R. (2022). Existing evidence on antibiotic resistance exposure and transmission to humans from the environment: A systematic map. Environ. Evid..

[29] Hart A., Warren J., Wilkinson H., Schmidt W. (2023). Environmental surveillance of antimicrobial resistance (AMR), perspectives from a national environmental regulator in 2023. Eurosurveillance.

[30] Hua M., Huang W., Chen A., Rehmet M., Jin C., Huang Z. (2020). Comparison of antimicrobial resistance detected in environmental and clinical isolates from historical data for the US. BioMed Res. Int..

[31] APHA (1998). Standard Methods for the Examination of Water and Wastewater.

[32] Turano A., Pirali F., Balows A., Hausler W.J., Ohashi M., Turano A., Lennete E.H. (1988). Quantification Methods in Microbiology. Laboratory Diagnosis of Infectious Diseases.

[33] Campoccia D., Montanaro L., Moriarty T.F., Richards R.G., Ravaioli S., Arciola C.R. (2008). The selection of appropriate bacterial strains in preclinical evaluation of infection-resistant biomaterials. Int. J. Artif. Organs.

[34] Centers for Disease Control and Prevention (2019). Antibiotic Resistance Threats in the United States, 2019.

[35] Durso L.M., Schmidt A.M. (2017). Antimicrobial resistance related to agricultural wastewater and biosolids. Antimicrobial Resistance in Wastewater Treatment Processes.

[36] Mulchandani R., Wang Y., Gilbert M., Van Boeckel T.P. (2023). Global trends in antimicrobial use in food-producing animals: 2020 to 2030. PLOS Glob. Public Health.

[37] Long N.S., Hales K.E., Berry E.D., Legako J.F., Woerner D.R., Broadway P.R., Carroll J.A., Burdick Sanchez N.C., Fernando S.C., Wells J.E. (2023). Antimicrobial Susceptibility of Trimethoprim–Sulfamethoxazole and 3rd-Generation Cephalosporin-Resistant *Escherichia coli* Isolates Enumerated Longitudinally from Feedlot Arrival to Harvest in High-Risk Beef Cattle Administered Common Metaphylactic Antimicrobials. Foodborne Pathog. Dis..

[38] Agga G.E., Schmidt J.W., Arthur T.M. (2016). Effects of in-feed chlortetracycline prophylaxis in beef cattle on animal health and antimicrobial-resistant *Escherichia coli*. Appl. Environ. Microbiol..

[39] (2015). Performance Standards for Antimicrobial Susceptibility Testing; Twenty-Fifth Informational Supplement. The β-Lactamase Disk Test: A Modified Method to Detect Extended-Spectrum-β-Lactamases in Multidrug-Resistant Escherichia coli Isolates..

[40] (2017). EUCAST Guidelines for Detection of Resistance Mechanisms and Specific Resistances of Clinical and/or Epidemiological Importance.

[41] Condon J.C., Lisa M. Durso 2025a. ESBL Antibiotic Susceptibility Disc Diffusion Test. protocols.io. https://www.protocols.io/view/esbl-antibiotic-susceptibility-disc-diffusion-test-eq2lyxpqrgx9/v1.

[42] Sanders E.R. (2012). Aseptic laboratory techniques: Plating methods. J. Vis. Exp..

[43] (2011). Real-Time PCR Detection of Shiga-Toxin Producing *Escherichia coli* (STEC), Serotype O157 and non-O157 in Fresh Produce and Food with non-O157 Isolation and Identification.

[44] Condon J.C., Lisa M. Durso 2025b. Standard Uniplex PCR SOP for Escherichia coli and Salmonella. protocols.io..

[45] Ducey T.F., Durso L.M., Ibekwe A.M., Dungan R.S., Jackson C.R., Frye J.G., Castleberry B.L., Rashash D.M., Rothrock M.J., Boykin D. (2020). A newly developed *Escherichia coli* isolate panel from a cross section of US animal production systems reveals geographic and commodity-based differences in antibiotic resistance gene carriage. J. Hazard. Mater..

[46] Doumith M., Day M.J., Hope R., Wain J., Woodford N. (2012). Improved multiplex PCR strategy for rapid assignment of the four major *Escherichia coli* phylogenetic groups. J. Clin. Microbiol..

[47] Clermont O., Bonacorsi S., Bingen E. (2000). Rapid and simple determination of the *Escherichia coli* phylogenetic group. Appl. Environ. Microbiol..

[48] Escobar-Páramo P., Grenet K., Le Menac’h A., Rode L., Salgado E., Amorin C., Gouriou S., Picard B., Rahimy M.C., Andremont A. (2004). Large-scale population structure of human commensal *Escherichia coli* isolates. Appl. Environ. Microbiol..

[49] Kolmogorov M., Yuan J., Lin Y., Pevzner P. (2019). Assembly of long error-prone reads using repeat graphs. Nat. Biotechnol..

[50] Schwengers O., Jelonek L., Dieckmann M.A., Beyvers S., Blom J., Goesmann A. (2021). Bakta: Rapid and standardized annotation of bacterial genomes via alignment-free sequence identification. Microb. Genom..

[51] Parks D.H., Imelfort M., Skennerton C.T., Hugenholtz P., Tyson G.W. (2014). Assessing the quality of microbial genomes recovered from isolates, single cells, and metagenomes. Genome Res..

[52] Ondov B.D., Treangen T.J., Melsted P., Mallonee A.B., Bergman N.H., Koren S., Phillippy A.M. (2016). Mash: Fast genome and metagenome distance estimation using MinHash. Genome Biol..

[53] Alcock B.P., Huynh W., Chalil R., Smith K.W., Raphenya A.R., Wlodarski M.A., Edalatmand A., Petkau A., Syed S.A., Tsang K.K. (2023). CARD 2023: Expanded Curation, Support for Machine Learning, and Resistome Prediction at the Comprehensive Antibiotic Resistance Database. Nucleic Acids Res..

[54] Grant J.R., Enns E., Marinier E., Mandal A., Herman E.K., Chen C.Y., Graham M., Van Domselaar G., Stothard P. (2023). Proksee: In-depth characterization and visualization of bacterial genomes. Nucleic Acids Res..

[55] OIE World organization for Animal Health (2021). OIE List of Antimicrobial Agents of Veterinary Importance. https://www.woah.org/app/uploads/2021/06/a-oie-list-antimicrobials-june2021.pdf.

[56] United States Food and Drug Administration (2023). 2023 Summary Report on Antimicrobials Sold or Distributed for Use in Food-Producing Animals. https://www.fda.gov/animal-veterinary/antimicrobial-resistance/2023-summary-report-antimicrobials-sold-or-distributed-use-food-producing-animals.

[57] Jacob M.E., Keelara S., Aidara-Kane A., Matheu Alvarez J.R., Fedorka-Cray P.J. (2020). Optimizing a screening protocol for potential extended-spectrum β-lactamase *Escherichia coli* on MacConkey agar for use in a global surveillance program. J. Clin. Microbiol..

[58] Arnold K.E., Laing G., McMahon B.J., Fanning S., Stekel D.J., Pahl O., Coyne L., Latham S.M., McIntyre K.M. (2024). The need for One Health systems-thinking approaches to understand multiscale dissemination of antimicrobial resistance. Lancet Planet. Health.

