# Supplementary material for: Agriculturally Sourced Multidrug-Resistant Escherichia coli for Use as Control Strains
Source: Pathogens. 2025 Apr 25;14(5):417. doi: 10.3390/pathogens14050417 (PMC12114057; doi:10.3390/pathogens14050417)
Supplement: Supplementary file 1 [file pathogens-14-00417-s001.zip › ★Control Strain Supplementary File 1 - Genome Maps.pptx]

## Slide 1
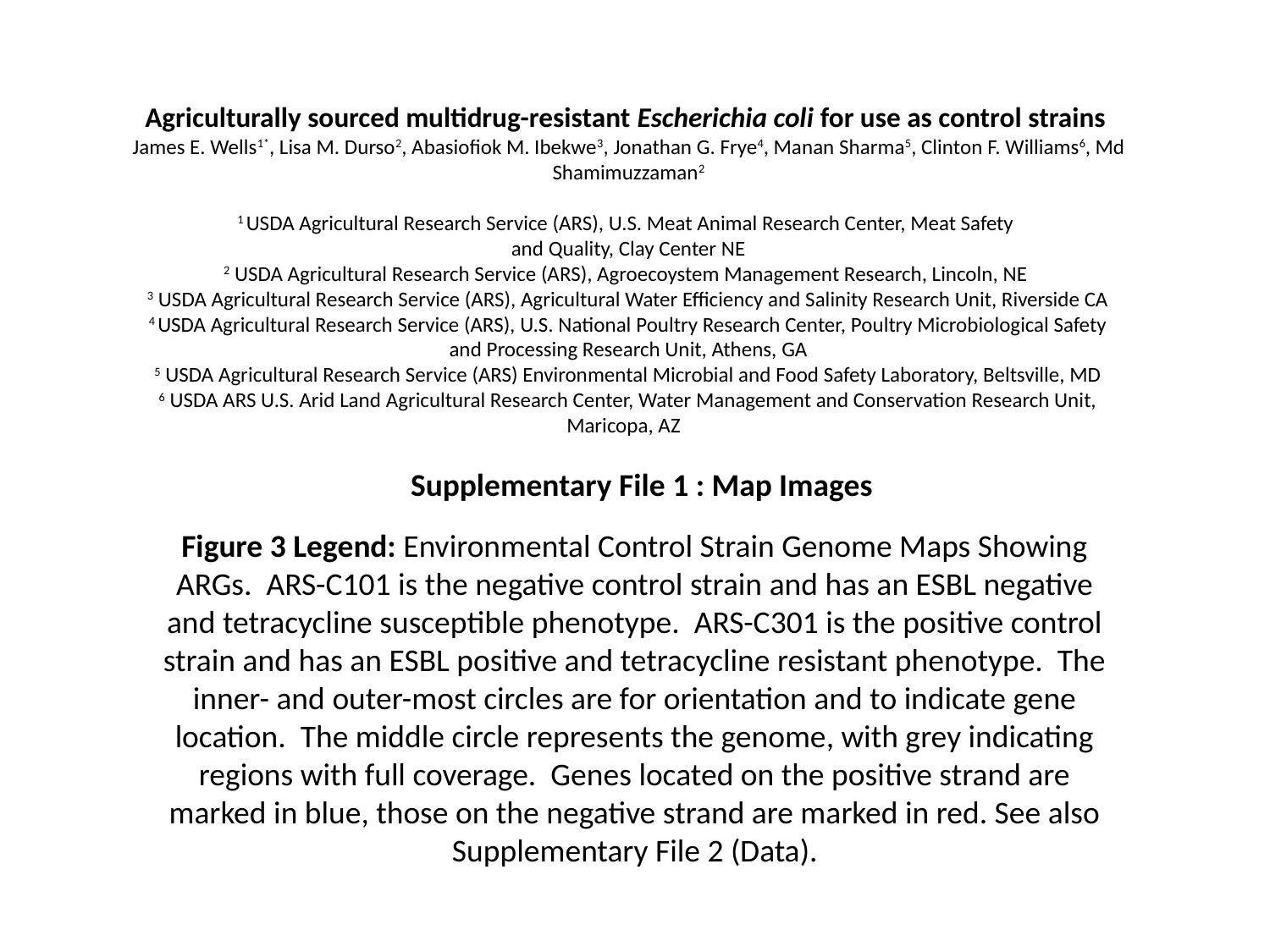

Agriculturally sourced multidrug-resistant Escherichia coli for use as control strains
James E. Wells1*, Lisa M. Durso2, Abasiofiok M. Ibekwe3, Jonathan G. Frye4, Manan Sharma5, Clinton F. Williams6, Md Shamimuzzaman2
1 USDA Agricultural Research Service (ARS), U.S. Meat Animal Research Center, Meat Safety
and Quality, Clay Center NE
2 USDA Agricultural Research Service (ARS), Agroecoystem Management Research, Lincoln, NE
3 USDA Agricultural Research Service (ARS), Agricultural Water Efficiency and Salinity Research Unit, Riverside CA
4 USDA Agricultural Research Service (ARS), U.S. National Poultry Research Center, Poultry Microbiological Safety and Processing Research Unit, Athens, GA
5 USDA Agricultural Research Service (ARS) Environmental Microbial and Food Safety Laboratory, Beltsville, MD
6 USDA ARS U.S. Arid Land Agricultural Research Center, Water Management and Conservation Research Unit, Maricopa, AZ
Supplementary File 1 : Map Images
Figure 3 Legend: Environmental Control Strain Genome Maps Showing ARGs. ARS-C101 is the negative control strain and has an ESBL negative and tetracycline susceptible phenotype. ARS-C301 is the positive control strain and has an ESBL positive and tetracycline resistant phenotype. The inner- and outer-most circles are for orientation and to indicate gene location. The middle circle represents the genome, with grey indicating regions with full coverage. Genes located on the positive strand are marked in blue, those on the negative strand are marked in red. See also Supplementary File 2 (Data).

## Slide 2
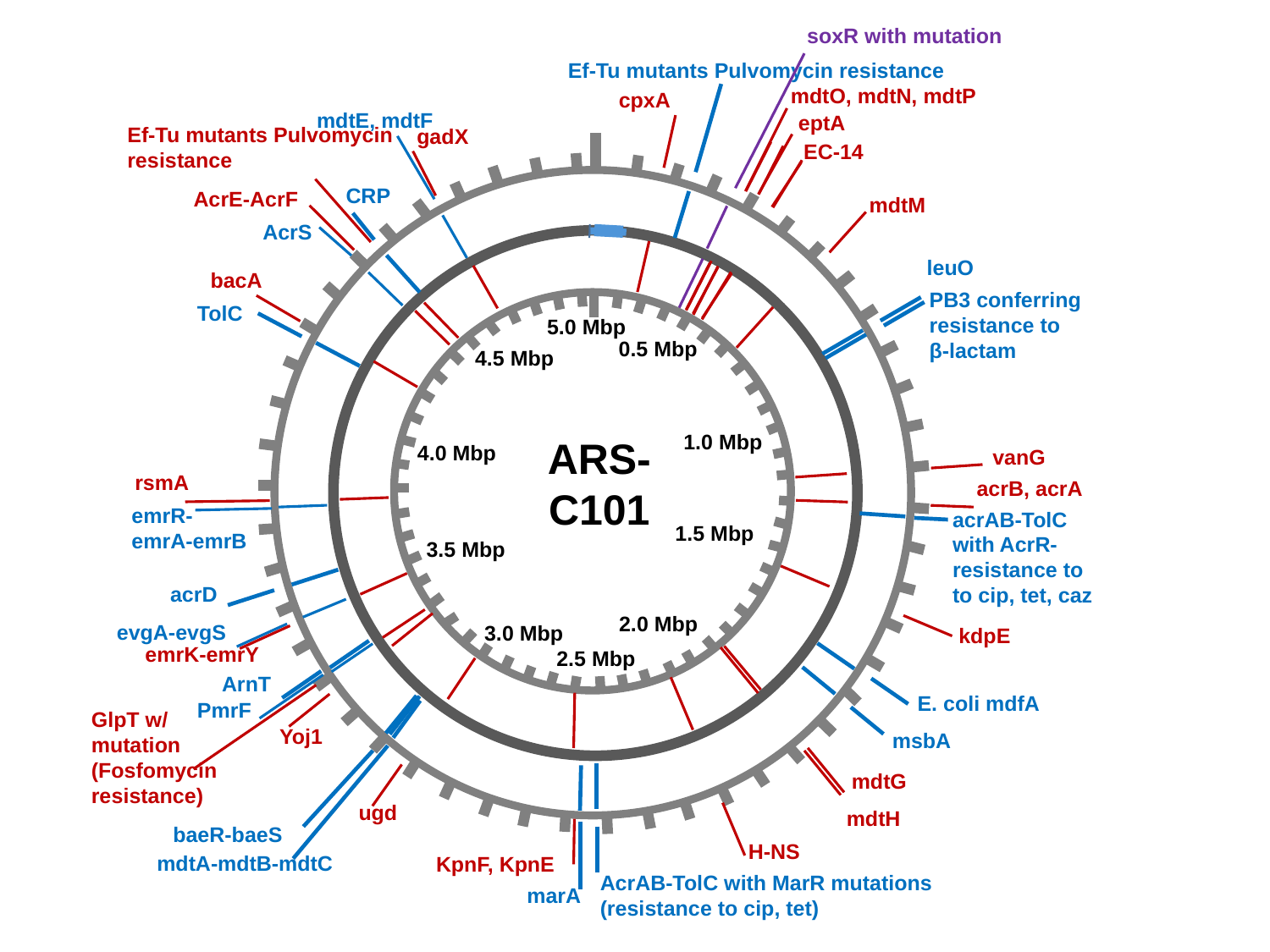

soxR with mutation
Ef-Tu mutants Pulvomycin resistance
mdtE, mdtF
CRP
AcrS
leuO
PB3 conferring resistance to
β-lactam
TolC
emrR-
emrA-emrB
acrAB-TolC
with AcrR- resistance to
to cip, tet, caz
acrD
evgA-evgS
ArnT
E. coli mdfA
PmrF
msbA
baeR-baeS
mdtA-mdtB-mdtC
AcrAB-TolC with MarR mutations (resistance to cip, tet)
marA
mdtO, mdtN, mdtP
cpxA
eptA
Ef-Tu mutants Pulvomycin resistance
gadX
EC-14
AcrE-AcrF
mdtM
bacA
vanG
rsmA
acrB, acrA
kdpE
emrK-emrY
GlpT w/
mutation (Fosfomycin resistance)
Yoj1
mdtG
ugd
mdtH
H-NS
KpnF, KpnE
5.0 Mbp
0.5 Mbp
4.5 Mbp
1.0 Mbp
4.0 Mbp
1.5 Mbp
3.5 Mbp
2.0 Mbp
3.0 Mbp
2.5 Mbp
ARS-C101

## Slide 3
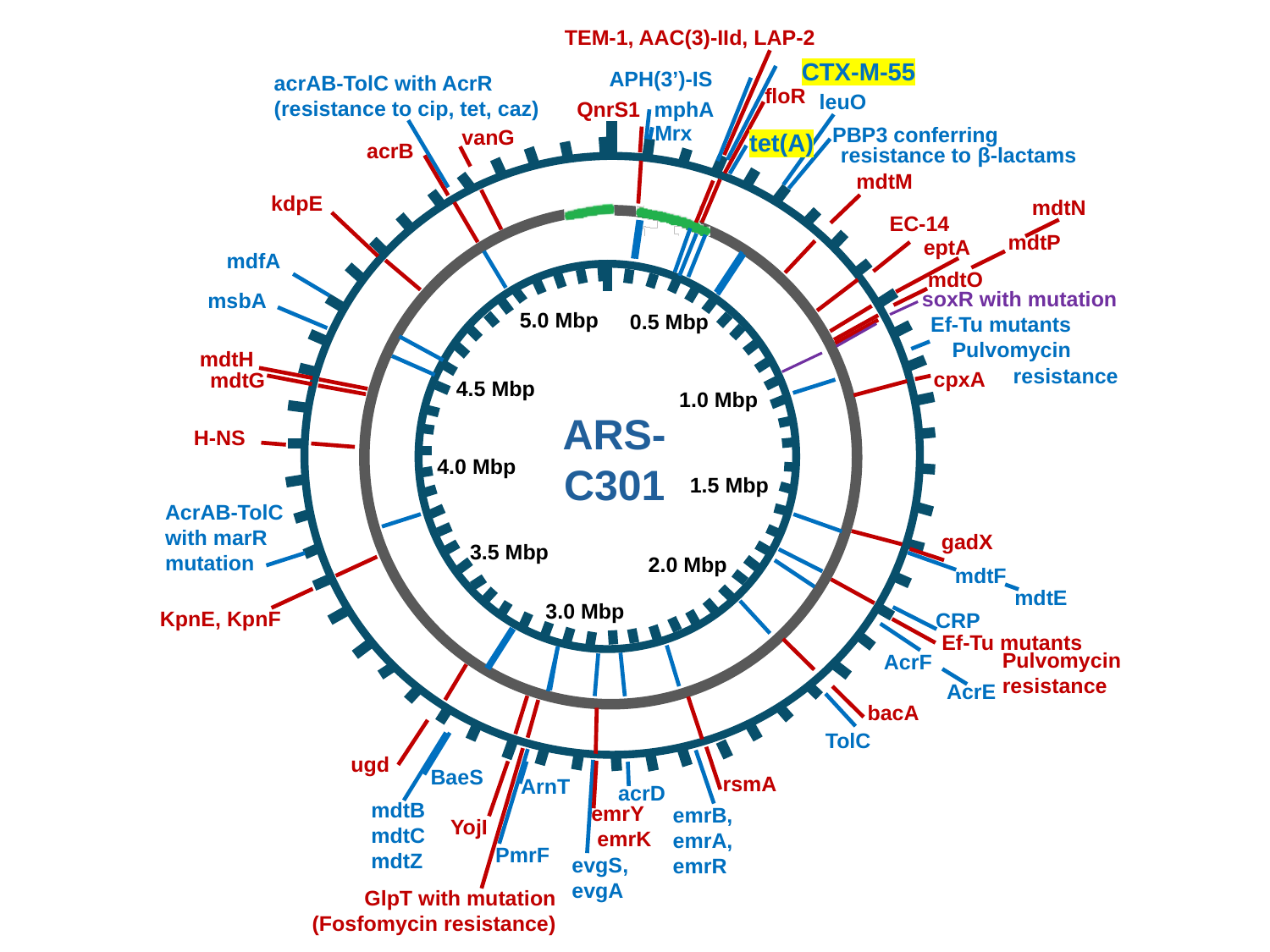

TEM-1, AAC(3)-IId, LAP-2
CTX-M-55
APH(3’)-IS
acrAB-TolC with AcrR (resistance to cip, tet, caz)
floR
leuO
mphA
QnrS1
Mrx
PBP3 conferring
resistance to β-lactams
vanG
ARS-C301
5.0 Mbp
0.5 Mbp
4.5 Mbp
1.0 Mbp
4.0 Mbp
1.5 Mbp
3.5 Mbp
2.0 Mbp
3.0 Mbp
tet(A)
acrB
mdtM
kdpE
mdtN
EC-14
mdtP
eptA
mdfA
mdtO
soxR with mutation
msbA
Ef-Tu mutants Pulvomycin
mdtH
resistance
cpxA
mdtG
H-NS
AcrAB-TolC with marR mutation
gadX
mdtF
mdtE
KpnE, KpnF
CRP
Ef-Tu mutants
Pulvomycin resistance
AcrF
AcrE
bacA
TolC
ugd
BaeS
rsmA
ArnT
acrD
mdtB mdtC mdtZ
emrB, emrA, emrR
emrY
 emrK
YojI
PmrF
evgS, evgA
GlpT with mutation (Fosfomycin resistance)

## Slide 4
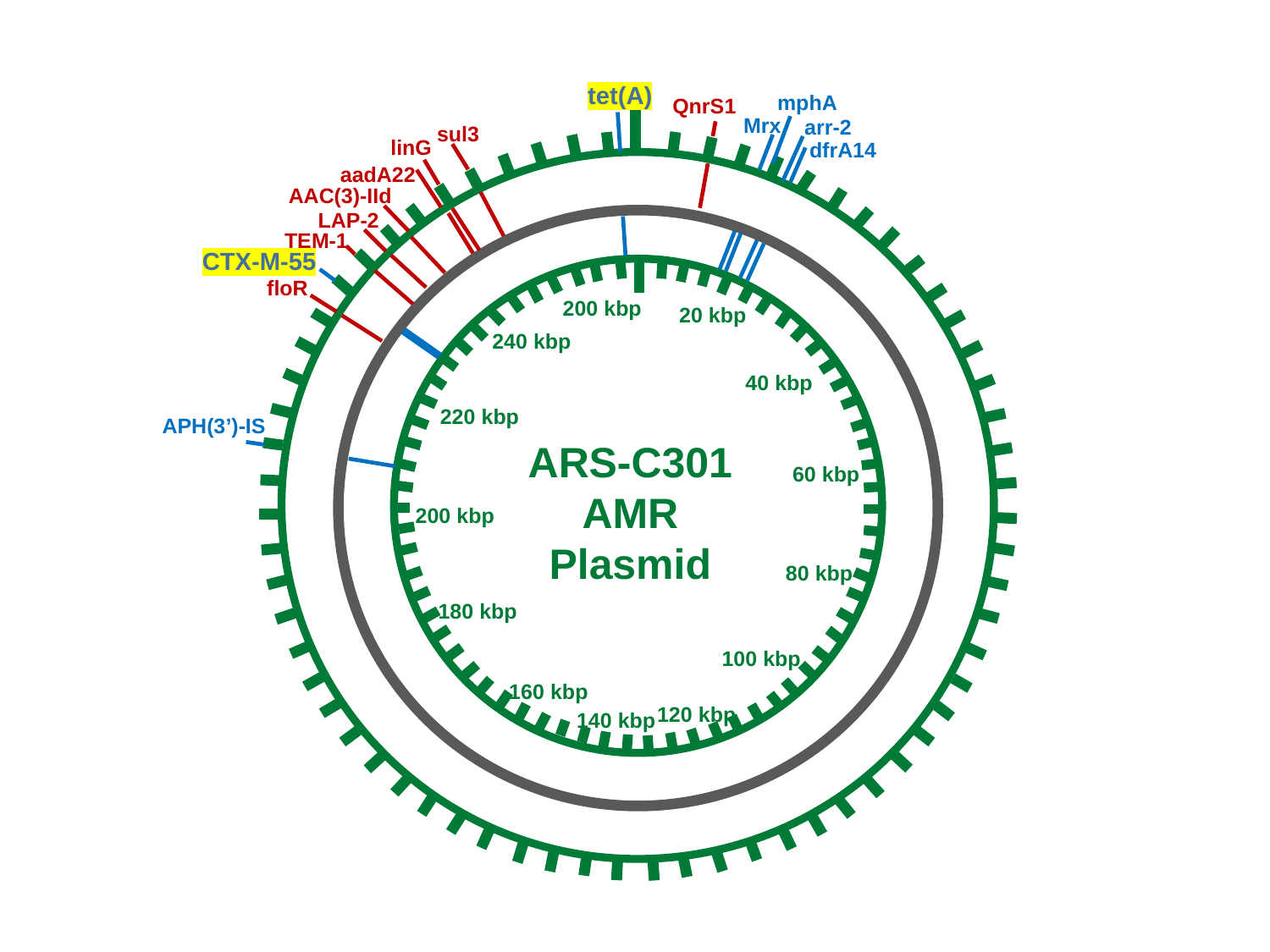

tet(A)
mphA
QnrS1
ARS-C301 AMR Plasmid
200 kbp
20 kbp
40 kbp
220 kbp
60 kbp
200 kbp
180 kbp
100 kbp
120 kbp
240 kbp
80 kbp
160 kbp
140 kbp
Mrx
arr-2
sul3
linG
dfrA14
aadA22
AAC(3)-IId
LAP-2
TEM-1
CTX-M-55
floR
APH(3’)-IS
